# Co-producing research on psychosis: a scoping review on barriers, facilitators and outcomes

**DOI:** 10.1186/s13033-023-00594-7

**Published:** 2023-08-30

**Authors:** C. E. Jakobsson, E. Genovesi, A. Afolayan, T. Bella-Awusah, O. Omobowale, M. Buyanga, R. Kakuma, G. K. Ryan

**Affiliations:** 1 Department of Psychiatry, Sussex Partnership NHS Foundation Trust, Eastbourne, England UK; 2 Department of Psychology, Institute of Psychiatry, Psychology and Neuroscience, King’s College London, London, England UK; 3 Centre for Child and Adolescent Mental Health, College of Medicine, University of Ibadan, Ibadan, Nigeria; 4 Department of Psychiatry & Centre for Child and Adolescent Mental Health, College of Medicine, University of Ibadan, Ibadan, Nigeria; 5 Department of Community Medicine, College of Medicine, University of Ibadan, Ibadan, Nigeria; 6 SUCCEED Africa, University of Zimbabwe, Harare, Zimbabwe; 7 London School of Hygiene and Tropical Medicine, Centre for Global Mental Health, London, England UK

**Keywords:** Co-production, Participatory research, Service user involvement, Psychosis, Schizophrenia

## Abstract

**Introduction:**

Co-production is a collaborative approach to service user involvement in which users and researchers share power and responsibility in the research process. Although previous reviews have investigated co-production in mental health research, these do not typically focus on psychosis or severe mental health conditions. Meanwhile, people with psychosis may be under-represented in co-production efforts. This scoping review aims to explore the peer-reviewed literature to better understand the processes and terminology employed, as well as the barriers, facilitators, and outcomes of co-production in psychosis research.

**Methods:**

Three databases were searched (MEDLINE, EMBASE, PsycINFO) using terms and headings related to psychosis and co-production. All titles, abstracts and full texts were independently double-screened. Disagreements were resolved by consensus**.** Original research articles reporting on processes and methods of co-production involving adults with psychosis as well as barriers, facilitators, and/or outcomes of co-production were included. Data was extracted using a standardised template and synthesised narratively. Joanna Briggs Institute and the AGREE Reporting Checklist were used for quality assessment.

**Results:**

The search returned 1243 references. Fifteen studies were included: five qualitative, two cross-sectional, and eight descriptive studies. Most studies took place in the UK, and all reported user involvement in the research process; however, the amount and methods of involvement varied greatly. Although all studies were required to satisfy INVOLVE (2018) principles of co-production to be included, seven were missing several of the key features of co-production and often used different terms to describe their collaborative approaches. Commonly reported outcomes included improvements in mutual engagement as well as depth of understanding and exploration. Key barriers were power differentials between researchers and service users and stigma. Key facilitators were stakeholder buy-in and effective communication.

**Conclusions:**

The methodology, terminology and quality of the studies varied considerably; meanwhile, over-representation of UK studies suggests there may be even more heterogeneity in the global literature not captured by our review. This study makes recommendations for encouraging co-production and improving the reporting of co-produced research, while also identifying several limitations that could be improved upon for a more comprehensive review of the literature.

**Supplementary Information:**

The online version contains supplementary material available at 10.1186/s13033-023-00594-7.

## Introduction

The involvement of service users in mental health research is increasingly recognised as best practice [1–3]. A previous review by Jo Brett and colleagues [4] identifies numerous positive outcomes of user involvement in health and social care research, across all stages of the research process [4]. In the early stages of agenda-setting and research planning, these include: identification of new research gaps and generation of further research questions, ideas, designs or proposals; improvement in the cultural equivalence of research tools and ethical considerations of trial design; and even the development of new medications. In terms of methodology and data collection: better application of the concept of informed consent and interpretation of information for participants; improved study design, including decision-making on endpoints, time of recruitment and selection of outcome measures; and more sensitivity to the general research climate. During write-up, dissemination and the implementation of results: opportunities to voice concerns about the interpretation of results and how implementation might be affected; and additional assistance in disseminating results to key stakeholders.

**Fig. 1:**
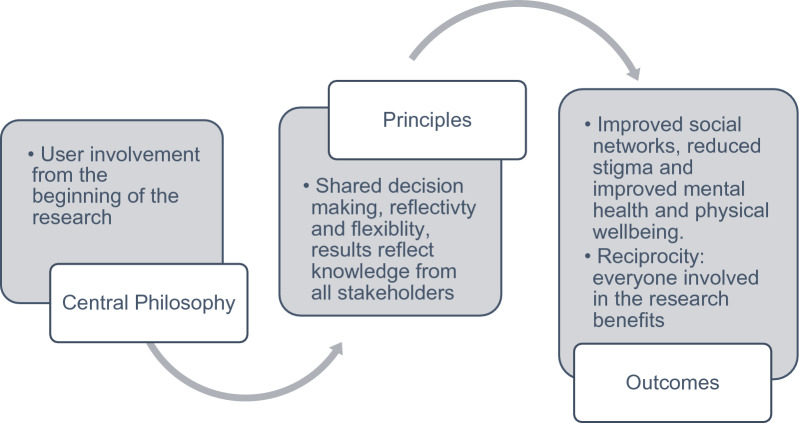
Co-production: philosophy, principles and outcomes [6, 9–11]

However, the definition of service user involvement is broad, and examples can range from tokenistic efforts, to sharing all decision-making and control, to entirely user-led research [5]. Indeed, Brett et al. [4] highlight that much of the research that claims to involve service users is limited to single-stage consultation, whilst user-led or collaborative efforts are more likely to promote involvement across all stages of the research process [4]. Co-production is one approach to collaboration “in which researchers, practitioners and the public work together, sharing power and responsibility from the start to the end of the project, including the generation of knowledge” (INVOLVE 2018, p.1) [6] (see Fig. 1 for summary of co-production process). The arguments for co-production typically fall into four categories: substantive (improving the quality of research), instrumental (greater impact), normative (related to the accountability of funders, addressing discourse between civic or public), and political (equality) [7]. With various stakeholders involved in the research process, co-production is particularly valuable as a tool to improve the relevance of and buy-in to academic research [8].

Although there are several frameworks and guidelines for co-producing research, there is no single formula for doing it, and this approach is not always straightforward. Perhaps as a result, researchers have been criticised for using the term “co-production” loosely to encompass a variety of different approaches to user involvement, some of which do not necessarily promote an equal sharing of responsibility between service users and researchers throughout all stages of the research cycle. Several barriers to co-production have also been identified. For example, co-production is often time- and resource-intensive, requiring relationship-building among diverse stakeholders—and the requisite exchange of favours and conflict resolution often involved in relationship management—with no guarantee of a good outcome [7]. Even where the co-production of research is successful, the translation of this research into practice can easily be derailed by poor organisation and planning, resulting in the disappointment and dissatisfaction of stakeholders involved [12, 13].

Some groups also have more opportunities for involvement in research than others. In particular, people with severe mental health conditions like psychosis may be under-represented in co-produced research. Psychosis is an overarching term for a number of symptoms, including hallucinations, delusions and thought disorder, which characterise several mental health disorders, such as schizophrenia [14]. A review by Woodall et al. identified several barriers to participation in mental health research among people with schizophrenia: fear, misunderstanding and mistrust of research, the medical establishment and medical interventions; the burden of participation coupled with insufficient remuneration, logistical difficulties and competing obligations; stigma, isolation and general lack of motivation; and severity of symptoms [15]. Any of these barriers could equally apply to co-production, which requires an even more intensive level of engagement than participation as a research subject.

### Rationale

While previous systematic reviews have investigated co-production in mental health research [3, 11], the transferability of their conclusions to research involving people with psychosis may be limited. This scoping review aims to explore the peer-reviewed literature as a starting point to develop more robust methods for systematically reviewing and appraising co-produced research on psychosis. The specific objectives are to document the terminology, methods and outcomes reported, as well as the barriers and facilitators to successful co-production in this context.

## Methods

### Eligibility criteria

Table 1 summarises our eligibility criteria for this scoping review. We included original research studies on psychosis (i.e., not protocols, reviews, commentaries, etc.) published in peer-reviewed journals that involved adults with psychosis, regardless of study design. Where study participants and/or the service users involved in the conduct of the research included a mix of adults and adolescents, we excluded studies where the mean age was under 18 years. As this is a scoping review, we employed a broad definition of psychosis. We primarily used the Diagnostic and Statistical Manual of Mental Disorders, Fifth Edition (DSM-5) criteria for Schizophrenia Spectrum and Other Psychotic Disorders and the International Classification of Diseases, Tenth Revision (ICD-10) definition of psychosis, which includes schizophrenia, schizotypal and delusional disorders. However, we also included studies where individuals were not directly diagnosed with a psychotic disorder, but were reported as having key symptoms such as delusions, hallucinations or thought disturbances, etc. In addition, we considered studies that failed to report on diagnosis or symptoms, so long as the studies themselves were investigating psychosis and we could reasonably infer from the text that at least some of the users involved came from this clinical population.

**Table 1:** Summary of inclusion and exclusion criteria

Criterion	Included
Study design	Original research on psychosis using any design (qualitative, quantitative, mixed-methods), with a co-production approach
Participants*	Adults (mean age 18 years or older) with reasonable expectation of lived experience of psychosis (based on diagnosis, symptoms, or other indications from text)
Reporting	Report details methods of co-production, plus one or more of the following: a) barriers and facilitators to co-production b) outcomes of co-production
Publication type	Published in a peer-reviewed journal

^*^Same criteria applied to research subjects as well as participants involved in co-produced aspects of research

We included papers in which the authors outlined a process of co-production and reported on barriers or facilitators to co-production, and outcomes of co-producing research. We anticipated that terminology and methodology might vary in practical application and therefore used a broad definition for co-production, characterised by the involvement of service users across multiple stages of the research process, starting from the beginning of the study. This allowed for a greater number of potentially relevant studies to be considered. We included papers that explicitly described methods of co-producing research on psychosis. Where this was unclear (for example, where a study did not describe its approach as “co-production” or similar), we referred to the five INVOLVE (2018) principles to make our screening decision: sharing of power, including all perspectives and skills, respecting and valuing the knowledge of all those working together on the research, reciprocity and building and maintaining relationships (see Additional file 4: Appendix D).

### Information sources and search strategy

The search strategy was developed with oversight from qualified librarians at King’s College London and the London School of Hygiene and Tropical Medicine. Search terms included subject heading and string searches for concepts related to psychosis and co-production, respectively (see Additional file 1: Appendix A for PsycINFO search terms). Subject headings were tailored for each of the three databases searched: PsycINFO, MEDLINE and EMBASE [16–18]. The literature search was limited to studies published in English. No other restrictions were applied. Searches were conducted on 24th June 2020.

### Screening and study selection

**Fig. 2:**
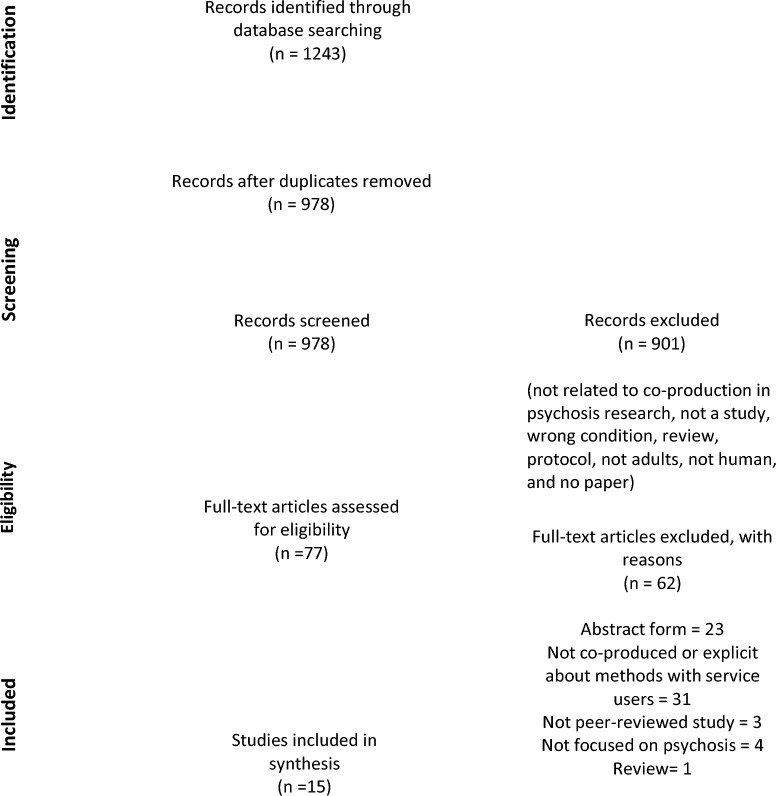
PRISMA Flow Diagram

Results from each database were exported into EndNote X9 [19], where duplicate studies were identified and deleted. Title, abstract and full-text screenings were completed independently by 2 screeners using the Rayyan web platform [20], and any discrepancies in screening were resolved by consensus. The four-phase PRISMA diagram summarised the study selection process (Fig. 2) and the PRISMA checklist was completed (Additional file 5: Appendix E).

### Data extraction, analysis and interpretation

Data was collected using a data extraction form covering participant and study characteristics and participatory research terminology, methods and outcomes (See Additional file 2: Appendices B, Additional file 3: Appendix C). While the INVOLVE (2018) principles were used to gauge initial eligibility during the screening phase [6], these include general, broad recommendations that are challenging to evaluate critically; hence, INVOLVE’s key features were used during data extraction and synthesis to assess further the extent to which service users were reportedly involved in the research process [6]. The eight key features address factors like continuous reflection, joint ownership of key decisions and a commitment to building relationships (see Additional file 4: Appendix D for the complete list). Each of these features was double-rated as fully present, partially present, not present, or impossible to determine from the included text (see Table 2).

**Table 2:**
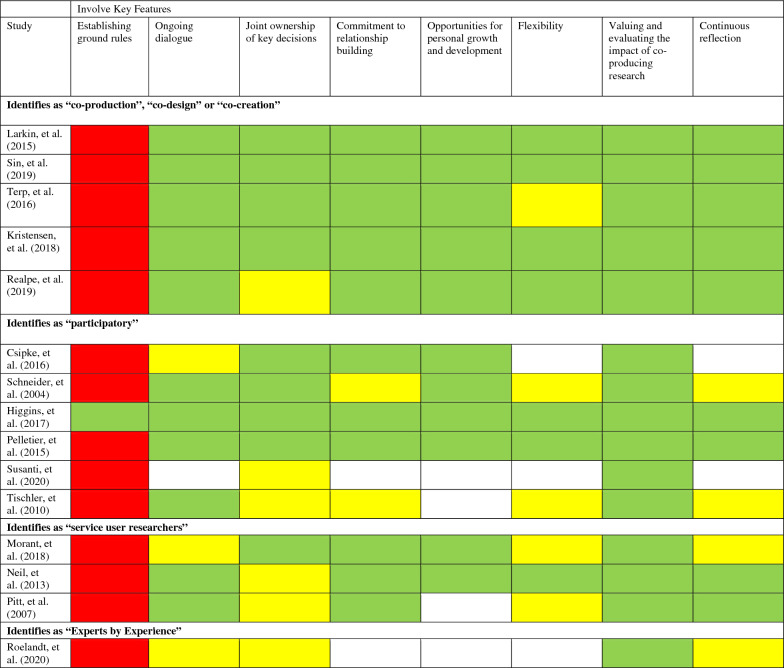
INVOLVE key features

Key: Partial = Yellow, Red = No, Green = Yes, Blank = Unclear

Results were synthesised following Popay et al.’s (2006) guidance on narrative synthesis by summarising, tabulating and grouping findings, then describing these narratively [21]. To aid in the interpretation of findings and draw conclusions and recommendations for co-production, co-authors from diverse perspectives (e.g., high-income country vs. low-/middle-income country, female vs. male, professional vs. lived experience of psychosis) participated in a series of six virtual discussion sessions and provided critical input into manuscript drafts.

### Quality appraisal

The quality of the included qualitative and cross-sectional studies were assessed using the Joanna Briggs Institute (JBI) Tools [22–24], and in the absence of an appropriate JBI tool we evaluated descriptive studies using the AGREE (Appraisal of Guidelines for Research and Evaluation in Europe) guidelines [25] (see Table 3). All the quality appraisals were completed independently by two reviewers, and any discrepancies were resolved by consensus.

**Table 3:** Co-production Components

References	Terminology	Methods	Barriers	Facilitators	Outcomes/and Conclusions
Identifies as “co-production”, “co-design” or “co-creation”					
Larkin et al. [31]	Evidence based Co-Design (EBCD)	Provided feedback to reach consensus during co-design, film shown and participants co-designed service improvements	Barriers: Censoring and safeguarding, stigma, high turnover, “lack of continuity in high-level support”	Facilitators: Feedback groups, formative qualitative research phase, “mechanisms to encourage ownership of the project, as well as strong high-level support, are essential to guarantee the implementation and sustainability of improvements”	“All feedback groups reached consensus fairly easily and recognized the typical difficulties faced by young service-users being hospitalized” “Attendance at the steering group was consistent among a small group of committed staff, but there was frustration at the lack of time and organizational support available to follow through the plans” Authors conlcude “ it necessary to develop and adapt the method to suit the vulnerable populations and complex services we were working within, but we feel able to conclude that it is feasible to use EBCD in a mental health context, provided careful attention is given to ethical and safety issues”
Sin et al. [28]	Coproduction, Participatory research methodologies	Co-design “workshops with an Expert Advisory Group” and “iterative consultations with carers”	Barriers: “Unintended pressure”	Facilitators: “Testimonials from other carers” and photos of EAG [Expert Advisory Group] working together”, “nondigital design tools” “encouraged creative design atmosphere”, “focus group consultation, and data analysis were performed in parallel with one another”	Authors commented that a significant outcome was the “documentation of a rigorous and innovative build process”
Terp et al. [30]	Co-design	Co-design workshops to “establish participation”	None Identified	Facilitators: Use of metaphors, enhanced communication “sense of community”: a community of practice inspired participation and fostered engagement, setting, support active participation (e.g., using storyboards)	Initial phase of design development created “ownership and pride” Advantage of experienced participant-facilitated “construction and maintenance of a secure and informal environment that supports mutual engagement”
Kristensen et al. [26]	Iterative co-creation process	Patient Peer Board “operated through workshops, on their own, in pairs and in groups to reach consensus recommendations.” Patient Peer Board comment on the “data collection”, “patient’s information requirements when answering questions related to PROMs”, “graphical display of PROMs”, and “graphical display of PRO results for self-management”	None Identified	Facilitators: Iterative co-creation and consensus process facilitated conflict resolution. Organization of study allowed patients to be “directly and indirectly involved at all project stages and voice their opinions” “Direct patient involvement is likely to build trust”	“Task of the PPB was to voice the patients’ opinions” “Emphasis on direct patient involvement is likely to build trust that the PRO-data will not be misused” Patient Peer Board successfully “emphasized concrete, unambiguous easily understandable information, and procedures for data collection and display of results”
Realpe et al. [32]	Co-design	Service users with experience of mental health services were invited to partake in a five-stage interactive co-design process Service users’ suggestions were used “to advance to the next stage.” Service users partook in different activities e.g. workshops, video screenings and provided feedback on the design and delivery on the online intervention platform	Barrier: Feedback groups “required considerable time and effort” and were impacted by lack of continuity “Lack of opportunities and support systems to facilitate collaboration between young people and researchers in developing research together” Access to technology	Facilitators: Clarity about “security of data storage”, as “this may be of particular issue for people with a history of paranoia/psychosis.”	“Participants in this study challenged researchers' understanding of what young people need during recovery” Findings contribute to current efforts to develop environments that reflect lived experience “The process of co- design led to the development of a specific approach and protocol to be tested in a proof-of-concept trial with people experiencing a first episode of psychosis” " Service users supported novel methods of engagement of people who may be suffering in isolation and found virtual worlds to delivery therapy acceptable” “The co-design process permitted a feedback loop that continues to inform design and solve problems as they emerge in the pilot study”
Identifies as “participatory”					
Csipke et al. [34]	Participatory methodology with service user researcher	Follows principles of ‘‘SURE” model Service user researchers involved throughout process; “one service user researcher had experience of the service under investigation”	None Identified	Facilitators: Use of a camera makes participants feel involved in the research, mitigates “language barriers”, provides “in-depth understanding of service user experience”	Development of patient generated outcome measures with “user generated tools [..] may enable an in-depth assessment of the experience of in-patient care for service users and frontline staff alike”
Schneider, et al. [33]	Participatory research	Service users “chose the topic”, “method of data collection”, resulting in a “set of recommendations” and influenced dissemination of findings	Barriers: Need to account for a “range of abilities”	Facilitators: Multiple forms of result dissemination (e.g., theater presentation and academic journal article), “good communication”	“This research project empowered one small group of very marginalized people with schizophrenia to speak directly to psychiatrists and other mental health professionals about their treatment experiences and through this is contributing to change in how others with mental illnesses are treated by their health care professionals”
Higgins et al. [27]	Participatory action research	Coordinated by a steering group, “core values were agreed by the group at the onset”, discussions held with service users and family members to gain their views on the program Designed education program and co-facilitated delivery of the education program	Barriers: Absence of literature on co-facilitation	Facilitators: Clinicians invited to discussions in order to generate ‘buy-in’, additional “consultation meeting held with multidisciplinary teams”	“Flexibility of the interview would shed light on unexplored or previously unexamined perspectives, such as the challenges of peer-clinician co-facilitation, the value of peer involvement and of programme handbooks”
Pelletier et al. [35]	Participatory action research	“Equitable participation” between non-academic members and co-research team “Close collaboration and consultation between the Advisory Board and Co-Research Team was undertaken at all key stages of development”	Barriers: For effective implementation, “perceptions of incompetence, of dangerousness, and of permanent impairment that health care providers can have towards patients” must be challenged	Facilitators: “Active and visible participation of patients in small group sessions with health care providers” helped “generate a more positive image and to strengthen GPs confidence in engaging” with patients “Small group learning” enables “participants to develop problem-solving, interpersonal, presentation and communication skills that are difficult to develop in isolation and require feedback and interaction with other individuals.”	“The patient-centeredness of this participatory research was characterised by an enhanced level of patient commitment to this R&D process of the IGMA and its use” “Difficult communication and social distance have been identified in the literature as bar- riers to equitable access to primary care providers and this access can be improved through the use of a tool like the IGMA and the interactivity and collective social support that its use generates among small groups of patients”
Susanti et al. [29]	Patient and public involvement	Consultation events with local collaborators, who informed data collection methods Advisory group “consulted on all project components.”	Barriers: Negative past experiences, “lack of understanding”, stigma, resources and caregiving burden	Facilitators: “Sense of purpose” and "reintegrate into the community”, range of involvement activities	“Participants acknowledged a number of benefits of PPI including sharing burden, sharing skills and experiences, improving confidence, and combating stigma”
Tischler et al. [36]	Participatory research	Contributed to design, “with support from the study team”	Barriers: “Power differential that exists between researchers and service user”, service users’ “lack of research experience”, jargon	Facilitators: Support enables sharing of “views without feeling intimidated or patronized”	“Emphasizes the importance of the process of research in achieving genuine service user involvement. It highlights the potential benefits of collaborative projects for both health professionals and service users despite the need for substantial resources and commitment to undertake such research”
Identifies as “service user researchers”					
Morant et al. [39]	Service user researchers	Interviews developed in “collaboration with an established mental health service users group South Essex Service Research Group” “Integrated service user perspectives into early stages of the analysis process”	None Identified	Facilitators: Disclosures by service user researchers “enhanced rapport and openness”	“In depth exploration of service users views of their involvement in decision-making about antipsychotic medication. As well as revealing the impacts of taking antipsychotics, it highlights how experiences of medication decision-making are characterised for many by passivity, perceived limitations of choice, low levels of involvement, and a sense of powerlessness”
Neil et al. [37]	Service user researchers	Supervised by qualified psychologist. Service users consulted with the Steering Committee (service users) throughout the research process	Barriers: Safeguarding Despite efforts to avoid “tokenistic involvement”, service users excluded from statistical analysis	Facilitators: Service users advised questionnaire; wording designed to “reduce the potential to cause distress”	“User with experience of psychosis, and some with experience of completing questionnaires. By working together and sharing our areas of expertise, for example, research knowledge and expertise by experience, we produced a recovery measure, which is meaningful and valid.”
Pitt et al. [38]	User-led research	“Research supervision provided by clinical psychologists” Service user researchers “met regularly with a steering committee” (service users) and “conducted all stages of the research”	Barriers: Setting, “independent representative”, “sufficient time”, respecting opinions, high “turnovers of staff” and meaningful therapeutic relationships	Facilitators: Service user researchers’ personal experience, empowerment, “broader ‘user perspective’” mitigates “personal bias from the primary investigators”	“Importance of promoting and encouraging active participation by service users and the need for strengthening key relationships with professionals who are able to engage in active dialogue with the service user”
Identifies as “Experts by Experience”					
Roelandt et al. [40]	Peer workers and experts by experience	“Users and carers were involved in all the stages of the project”; “participated in the development of the protocol, in the review of the design and the materials, in pretesting, and in the interpretation of the results.” Interviewers included “peer workers and experts by experience.”	Barriers: Disclosure Results and findings “may not be applicable to persons not informed of their diagnosis or outside of the health care system"	None Identified	“Revealed a gap between official medical language and users’ and carers’ language.” Experiential knowledge “could be a source of relevant information to reduce the gap between chosen terms and underlying concepts”

## Results

A total of 1243 records were identified through the database search. After duplicates were removed, 978 remained, and the titles and abstracts of these records were screened alongside the pre-specified eligibility criteria. This resulted in a decision to exclude 901 records. The remaining 77 records underwent full-text screening, with an additional 62 records excluded. Fifteen studies were included in this scoping review.

### Characteristics of included studies

#### Study designs

There was a mix of qualitative research (n = 5), descriptive studies (n = 8) and cross-sectional quantitative studies (n = 2). Several of the studies had multiple components to them and varied in sample size at each stage. The sampling strategy differed across the studies; however, two of the studies did not specify their sampling strategy, and four studies did not include any participant characteristics. Table 4 provides more detailed information on type of study and other key characteristics, where available.

**Table 4:** Study Characteristics

	Focus of the study					Co-production component		
Study	Country	Study Topic	Study type	Sample	Participant and/or Service Users Diagnosis	Research team	Steering group	Other roles e.g., participants
Csipke et al. [34]	England	“Service user and frontline staff perspectives on psychiatric ward design”	Descriptive Participatory Methodology (Adapted the “SURE Model”)	Purposive sampling of 20 service users (during interview phase) and 104 service users (during questionnaire phase) Mean age during measure development phase was 44.20, during the questionnaire phase 41.41 and during the photography phase 44.20	Schizophrenia/Psychosis, Bipolar Disorder, Depression/Anxiety, Substance Misuse, Dual Diagnosis (options for Other and Not Disclosed also provided)	Two service user researchers Staff (qualified nurse, student nurse, domestic or registrar and researchers)	NA	Service user as study participants (voluntary, under section or not disclosed/unavailable)
Higgins et al. [27]	Ireland	Development and evaluation of an “information programme for users of services and family members”	Descriptive Participatory Action Research Sequential Mixed Method Design	58 participants during the research phase (30 service users, 21 family members and 7 clinicians), 13 clinicians and 10 peer facilitators attended the initial training, and 30 service users attended the pilot programme; all aged between 23–80 and referred from a local mental health services	Schizophrenia, Bipolar Disorder, Schizoaffective Disorder, Severe Depression or Psychotic Depression	Clinicians and multidisciplinary clinical teams	Yes—“Steering group comprised on representatives from all stakeholder groups involved”	Service users and family members as study participants
Kristensen et al. [26]	Denmark	Consensus building on “patient-reported outcome measures” for “psychiatric clinical registries”	Descriptive Iterative co-creation process	30 service users (10 Patient Peer Board and 20 Steering Group) all “appointed by the two relevant stakeholder organizations” Age not reported	Depression and Schizophrenia	Patient Peer Board Members (PPB), Steering Group (SG) comprised of an interdisiplinary group of healthcare care professionars	Yes — SG	NA
Larkin et al. [31]	England	Service user, staff and parent perspective on improving the “experience of hospitalization during early psychosis”	Qualitative Experience Based Co-Design Action Research	150 participants recruited through purposive sampling Age not reported	Psychosis	Collaboration with service users, carers, community and impatient staff and managers	Yes—“Steering groups, which included NHS staff, service users and family members”	Feedback groups “ consisting separately of inpatient staff, community mental health staff, NHS managers, family members, or service-users”Servicer users, parents and inpatient staff as study participants
Morant et al. [39]	England	“User experiences of antipsychotic medication and lack of involvement in medication decisions”	Qualitative	20 participants recruited through purposive sampling mean age of 40.3	Schizophrenia and Schizoaffective Disorder	Service user research and study researchers	Yes—“collaboration with an established mental health service users group South Essex Service User Research Group (SE-SURG)”	Service users as study participants
Neil et al. [37]	England	Development and validation of a “measure of recovery from psychosis”	Descriptive	10 to 15 participants recruited through convenience sampling Age not reported	Psychosis	Two service user researchers, service user consultants, graduate research assistant, trainee clinical psychologist and qualified clinical psychologists	Yes—Steering committee “consisted of 10 and 15 Service Users”	NA
Pelletier et al. [35]	Canada	“Improve access to primary care for the physical health of patients with servere mental illnesses”	Quantitative Participatory Action Research Design	146 participants with a mean age of 52.7 recruited through a non-profit organisation	Schizophrenia	Co-research team including a physician with mental illness, psychiatrists, Director of the IPPAR(International Program for Participatory-Action Research), adjunct director of the local health authority, general practitioner, university professor of nursing, research assistant with mental illness	Yes—“2 patients were members of the Advisory Board that provided leadership and advice” as well as the “physician with mental illness, psychiatrists, director of the IPPAR, investigator familiar with Australian Guideliens, adjuct director of the local health authority, general practitioner, practicing nurse, family member (mother of a person with serious mental illness), communication specialist with health profssionals, univeristy professor of nursing, research assistant with mental illness, PhD candidate in nursing”	“Mental health service users who interact with study participants in small group learning sessions”IPPAR members include “psychican with mental illness”, “investigator familiar with the Austrlian Guidelines”, and “research asssistants with mental illness”
Pitt et al. [38]	United Kingdom	Experience of "people’s recovery from psychosis; to define recovery from a user perspective”	Qualitative	7 participants recruited through mental health groups aged 18–65	Psychosis	Led by two user researchers, in collaboration with a steering committee, with supervision from clinical psychologists	Yes—“Steering Committee of service users”	Service user as study participants
Realpe et al. [32]	United Kingdom	Co-design of an online platform “to deliver social cogntivie therapy in early psychosis”	Descriptive Co-design	Stage 1&2: 2 service users Stage 3: four service users Stage 4: four volunteers from target population but without mental health history Stage 5: 20 participants recovering from first episode psychosis Recruited through mental health partnerships and universities Age not reported	Psychosis	Service users and study researchers	Yes—Consulted MQ Young People’s Advisory Group	“Four university students” (non-service users) involved to “beta test the prototype”
Roelandt et al. [40]	15 countries^a^	Service user and carer perspectives “about “depressive episode” and “schizophrenia” diagnoses”	Mixed Method Participatory Research	263 service users and 255 carers recruited as the visited mental health services in study sites with a mean age of 39.4 for users and mean age of 55.1 for carers	Schizophrenia and Depression	Service users and carers (“part-time or full-time, on a personal basis and in a continuous manner”), “health care professionals working in the field of mental health, including peer workers and experts by expereince”	Yes—Steering committee	Service users and carers as study participants
Schneider et al. [33]	Canada	“Communication Between People with Schizophrenia and Their Medical Professionals”	Qualitative Participatory Research	11 participants, recruitment strategy not reported Age not reported	Schizophrenia	People with schizophrenia from the Unsung Heroes Peer Support Group were both the researchers and the research participants (e.g., service user researchers interviewed each other and analysed the data), university researcher and research assistant	NA	Service users as study participants
Sin et al. [28]	England	“Multicomponent eHealth Intervention for Family Carers for People Affected by Psychosis”	Descriptive Participatory Research Methodologies	24 participants with EAG recruited through various digital communications and carers from 3 mental health trusts all aged between 23 and 83	Psychosis or Schizophreniform	Expert advisory group (EAG) “comprising of individuals with lived experience of psychosis, carers, health care professionals, researchers, voluntary organization workers and elearning experts”	Yes—EAG Focus groups discussions with additional carers (who were independent from the study) were consulted on the drafts of the intervention	NA
Susanti et al. [29]	Indonesia	“Patient and public involvement to strengthen Indonesian mental health care for people with psychosis”	Qualitative	22 service users and 21 carers recruited through convenience sampling; with a mean age of 38.8 for user and mean age of 51.7 for carer	Psychosis	Patient and public involvement (PPI) group (service users, carers) mental health nurse academic, and carer-researcher	Yes—PPI advisory groups consisting of people with lived expereince of psychosis or carers	Service user and carer as study participants
Terp et al. [30]	Denmark	“Young adults with schizophrenia become strong Collaborators”	Descriptive Qualitative	7 young adults, 7 healthcare professionals recruited through purposive sampling aged between 19–31	Schizophrenia	Young adults with Scizophrenia, healthcare providers, software designers, graphic designers, graphic recorder and team leader	NA	Service users and health professionals as study participants
Tischler, et al. [36]	England	Patient- Centeredness	Mixed Methods Community Study	14 mental health service users and 3 senior psychiatrists were self-selected Age not reported	Schizophrenia	Service usrs and senior psychiatrists (one of the psychiatrist was also a mental health service user) and study team	NA	Service users and psychiatrists as study participants

^a^Algeria, Canada, France, Greece, Hungary, India, Italy, Lebanon, Lithuania, Madagascar, Mauritania, Mexico, Morocco, Spain, and UK

#### Study participants

Most of the studies occurred in high-income countries (most frequently within the United Kingdom), and only two studies included low- or middle-income countries (LMICs). In five of the 15 included studies, the participants’ ages were not specified; however, age ranges were indicated in other ways. For example, Kristensen et al. (2018) stated that the target group for the co-designed patient-reported outcome measure was patients 18 years or older [26].

#### Involvement of service users

Among the service users involved in the studies, the most common diagnosis was schizophrenia. Service users commonly had roles as members of an expert advisory group or “steering committee”. The collaborative research team was often comprised of service users, healthcare professionals, and academic researchers. A few studies involved service users both as part of the study team and as part of a steering group. Carers were also involved in three studies [27–29]. One study had a carer researcher in addition to a patient advisory group [29].

### Co-production components of included studies

The terminology used to describe user involvement varied significantly between the studies, though each of the included texts recognised the expertise that service users contribute to research. The methods of service user involvement employed also varied widely: four of the studies sought consensus from service users (participants and/or members of the research team), five consulted service users or service user reference/steering groups, three utilised collaborative workshops with service users, and four had service user researchers.

When the INVOLVE key features were applied to assess the level of co-production across the included studies, it was notable that all of the studies except Higgins et al. [27] failed to report ground rules at the onset of the research project. Roughly half of the studies discussed partial ownership of the research and reported a flexible process, which enhanced relationships and created opportunities for growth and development on the part of both the researchers and service users. All of the studies acknowledged the value of lived experience in research and most of the studies used continous reflections and ongoing dialogue to support an iterative process of collaboration. Table 2 assigns a colour-coded summary to each of the INVOLVE key features, indicating whether the feature is at least partially (yellow) or fully (green) present, not present (red), or whether it is impossible to determine from the texts (no colour). Table 2 further organises the included studies by the terminology used to describe the co-production approach (i.e., “co-”, versus “participatory”, “service user researchers”, “experts by experience”). These categories are also described narratively, below.

#### “Co-production”, “co-design” or “co-creation”

Although assessing their rigour is challenging, due to lack of methodological justification and data triangulation, the five studies included in this category described a long-term collaborative process referred to as either “co-production”, “co-design” or “co-creation”. These studies demonstrated a flexible approach with ongoing dialogue, continuous reflection, and a commitment to fostering relationships. However, none reported that ground rules were established at the onset of the research, even though this is one of the central features of co-produced research according to INVOLVE [6]. Sin et al. used participatory methodologies described alternately as co-production and co-design in their report, illustrating the interchangeability of terms used to describe user involvement [28]. The researchers used participatory design workshops, iterative consultations with stakeholders, and an Expert Advisory Group (EAG) to design an eHealth intervention. In each workshop, all members reportedly assumed equal decision-making roles and contributed their respective strengths and expertise. Similarly, Terp et al. aimed to improve co-design methods by conducting workshops so that young adults with schizophrenia could become active participants in mental health services [30]. Their workshops utilised various tools, including non-digital materials and metaphors, to encourage active participation. Larkin, Boden and Newton conducted experience-based co-design events to improve healthcare services by enabling service users, carers, and staff to collaborate [31]. Likewise, Kristensen et al. used an iterative co-design method, where the output was based on a co-creation process between patients and healthcare professionals [26]. The Patient Peer Board (PPB) participated in workshops and worked individually, in pairs, and in groups facilitated by an interdisciplinary steering group to reach a consensus. Finally, Realphe et al. invited service users to provide feedback on designing and delivering an online platform for early psychosis to encourage participation from hard-to-engage service users [32]. The authors used an iterative five-stage co-design process with service user involvement throughout all stages, in which the feedback from one session informed the next.

### “Participatory”

The six studies included in this category also addressed the INVOLVE [6] principles for co-production but identify their approach as “participatory research”. Higgins et al. included focus group discussions to gauge information regarding the co-design and co-facilitation of the education program. Core values were agreed upon at the project’s outset, making this the only study to report ground rules, and indeed the only to satisfy all eight key INVOLVE features. Schneider et al. also used a participatory research approach in which service users were involved throughout, yet only partially achieved several key INVOLVE criteria. The research team agreed that the findings would be disseminated through a theatrical performance as per the participants’ preferences and published in an academic journal to reach a larger audience [33]. The service users would be listed as co-authors but not involved in the writing of the research publication. The authors, however, acknowledged this project’s benefits in generating knowledge and offering a transformative experience for those involved.

Participatory research studies that involved service user researchers often achieved at least partially the INVOLVE features of joint ownership, personal growth, development, and ongoing dialogue, though there was not always sufficient information reported to make a full assessment. Csipke et al. referred to an adaptation of the “SURE’’ model, where service user researchers were involved in surveying the literature, performing the data collection, and undertaking a considerable part of the data analysis. One of the researchers had experience with the service under investigation, which provided greater insight [34]. Pelletier et al. employed participatory action research, which relied on close collaboration and consultation with an advisory board and the co-research team throughout all stages of the research [35]. The emphasis was on joint participation between non-academic members and the co-research team, where those involved immediately benefitted from the research and knowledge generation process. Additionally, the authors of the research publication included a physician with lived experience. Moreover, Tischler et al. [36] acknowledged the difficulty of establishing collaboration in practice due to the service users’ relative lack of research experience, which required support from the research team [36]. While service users contributed to the study design, they were also participants in focus groups, blurring the lines between research collaborator and research subject. Finally, Susanti et al. explored patient and public involvement (PPI) as a tool to strengthen the Indonesian health system [29]. The PPI advisory group consisted of individuals who either had lived experience of psychosis or cared for someone with a diagnosis of psychosis. Although the PPI advisory group was consulted throughout the entire research process, involvement was perhaps not sufficiently collaborative to qualify as co-production. One carer-researcher co-facilitated focus groups together with two mental health nurse academics and contributed to the analysis of transcripts. All the researchers were supervised by academics overseas in the United Kingdom.

### “Service user researchers”

Three studies employed service user researchers in their collaborative approaches and met a majority of the INVOLVE key features. Neil et al. described a previous study’s collaborative process that explored the relationship between service users and researchers; however, the researchers were also the supervisors, suggesting a potentially problematic power hierarchy [37]. Similarly, Pitt et al. used a steering committee consisting of service users who guided the study design and analysis from the commencement of the research [38]. Yet the project was overseen by clinical psychologists, as there was no service user researcher in a supervisory role, which might again suggest that power differentials existed between service user researchers and other researchers. During the analysis, the researchers’ interpretative influences on the data were mitigated by service user researchers who had lived experience and by input from the steering group. Morant et al. [39] utilised semi-structured interviews developed with a mental health service user group [39]. Service users conducted the interviews and disclosed their status as service users as part of rapport-building. Morant et al. [39] were aware of the existing preconceptions of academics, researchers and clinicians, and integrated service user perspectives into the early stages of the analysis process. Service users were invited to review, discuss and modify an initial set of emerging themes; however, the amount and impact of collaboration remains unclear. Although service user researchers conducted the study, much like Neil et al. [37] and Pitt et al. [38], the process still appeared to be guided by academics.

### “Experts by experience”

Although the final study explained that they involved ‘experts by experience’ in their research, they exhibited a minority of the INVOLVE features. Roelandt et al. stated that service users and carers partook in all stages of the research [40]. Although this aligns with co-production principles, it is not described further, and the extent to which users and carers participated may be limited.

#### Barriers and facilitators to co-produced research

Common barriers to co-production reported by the included studies were challenges with safeguarding [28, 37, 41], power imbalances [37, 39], mental health stigma [29, 31, 35], and high turnover among mental health professionals [31, 38]. Included articles highlighted that co-produced studies must pay extra consideration to recruitment strategies and study settings, aiming to mitigate barriers that may affect participants’ ability or willingness to partake in research, such as economic disadvantage or mistrust (for example of the Internet) [28, 32, 37, 41]. Another barrier identified was power differentials, namely where researchers maintain control of the process: for example, unilaterally deciding what materials to share from a data analysis and co-design event [37, 39]. Other limiting factors were existing preconceptions of user involvement and mental health stigma. For example, Susanti et al. reported patients’ past experiences with health professionals in which complaints during co-produced research were interpreted as a relapse in their condition [29]. Researchers can acknowledge these power imbalances and take active measures to ensure genuine collaboration. For instance, Tischler et al. [36] expressed the need for adequate support of service users when attending research meetings with other health professionals to empower service users to express their views [36]. As demonstrated in two studies, high turnover in mental health personnel can also impact the relationship between service users and health care professionals [31, 38]. As good communication and ongoing dialogue are essential facilitators for co-produced research, a critical barrier to the success of these projects emerged when support from personnel was discontinued [31].

Common facilitators of co-production reported by the included studies were stakeholder buy-in, effective communication [30, 33, 35], and additional support by providing and presenting materials in multiple formats and creating a safe and informal environment [28, 36]. For example, Sin et al. provided nondigital materials such as post-it notes and pens to facilitate a creative and inclusive design atmosphere [28]. This allowed all participants to engage in the co-design workshops, regardless of their technical abilities. Co-production was found to enable effective communication by offering the opportunity to resolve potential tensions throughout the process [26, 31] and empowering marginalised groups through genuine collaboration [26, 33, 38]. Further, as Realpe et al. explained, it is important that researchers communicate how the study data will be stored as this might be a particular concern for service users with a history of psychosis [32].

#### Outcomes

The included studies agreed that service user involvement in research provided greater insight by harnessing lived experience. Collaborative research approaches created a secure and informal environment, which encouraged mutual engagement and resulted in more in-depth understanding and exploration [26, 27, 30, 33, 36–39]. Pitt et al. explained that user-led research enabled strengthened relationships, facilitated communication, and created a unique rapport during user interviews, allowing for the collection of data that might not otherwise be accessible [38]. Involvement allowed service users to share personal experiences, knowledge and skills, and contribute to a flexible, collaborative and iterative approach [27, 32, 37, 40]. For example, Neil et al. acknowledged that the mix of knowledge and skills allowed for healthy debates and a regular review of topics; this was essential to ensuring their results were valid and meaningful [37].

Additionally, meaningful service user involvement addressed systemic barriers within mental health care by, for example, building skills, improving confidence and social connection, and combating stigma [29, 30, 33, 35]. As Terp et al. and Schneider et al. explained, increased ownership and pride amongst service users created a sense of meaning and purpose, and a collaborative research empowered marginalised individuals to participate in the process of change [33, 42].

### Robustness of results

All included studies reported positive outcomes of involving service users in the research process, indicating some degree of consensus within this body of research. However, the quality of the study designs and reporting, and particularly the lack of methodological justification, make it difficult to assess their rigour and call the robustness and generalisability of these studies into question (see Table 5 for details). For example, although it is generally accepted that qualitative studies tend to have smaller sample sizes, none of these studies transparently discussed how sample sizes were determined. Study reports often had missing components, including information regarding the researchers’ reflexivity, inclusion criteria for the participants, and sampling strategies. In specific instances, the broad spectrum of psychosis and potential for consensus bias [26, 31], issues with recruitment strategies [39], and problems with language translation [40] raised questions of validity. Reflecting the dynamic nature of collaborative research, several stages of research were often reported in a single paper, blending different techniques of analysis, which frequently lacked clear justification.

**Table 5:** Joanna Briggs Quality Appraisal Summary

Qualitative studies										
Reference	Congruity between stated philosophical perspective and the research methodology	Congruity between research methodology and research question or objectives	Congruity between the research methodology and methods used to collect data	Congruity between the research methodology and representation & data analysis	Congruity between the research methodology and the interpretation of results	Statement locating the researcher culturally or theoretically	Influence of researcher on the research, and vice- versa, addressed	Participants, and their voices, adequately represented	Ethical research according to current criteria and evidence of ethical approval by appropriate body	Conclusions drawn flow from the analysis, or interpretation, of the data
Larkin et al. [31]	Unclear	Yes	Yes	Unclear	Yes	No	Unclear	No	Yes	Yes
Morant et al. [39]	Unclear	Yes	Unclear	Unclear	No	No	Yes	Yes	Yes	Yes
Pitt, et al. [38]	Yes	Yes	Yes	Yes	Yes	No	Yes	Yes	No	Yes
Schneider et al. [33]	Unclear	Yes	Yes	Yes	Yes	No	Yes	Yes	Yes	Yes
Susanti et al. [29]	Yes	Yes	Yes	Yes	Yes	No	No	Yes	Yes	Yes

Cross-sectional studies								
References	Were the criteria for inclusion in the sample clearly defined?	Were the study subjects and the setting described in detail?	Was the exposure measured in a valid and reliable way?	Were objective, standard criteria used for measurement of the condition?	Were confounding factors identified?	Were strategies to deal with confounding factors stated?	Were the outcomes measured in a valid and reliable way?	Was appropriate statistical analysis used?
Roelandt et al. [40]	Yes	Yes	Unclear	Yes	Unclear	No	Yes	Yes
Tischler et al. [29]	No	Unclear	Unclear	Unclear	No	No	Unclear	Unclear

Descriptive studies								
References	Were patient’s demographic characteristics clearly described?	Was the patient’s history clearly described and presented as a timeline?	Was the current clinical condition of the patient on presentation clearly described?	Were diagnostic tests or assessment methods and the results clearly described?	Was the intervention(s) or treatment procedure(s) clearly described?	Was the post-intervention clinical condition clearly described?	Were adverse events (harms) or unanticipated events identified and described?	Does the case report provide takeaway lessons?
Csipke et al. [34]	Unclear	NA	NA	NA	Yes	NA	NA	Yes
Higgins et al., [27]	Yes	Unclear	Yes	NA	Yes	NA	NA	Yes
Kristensen et al. [26]	No	NA	NA	NA	Yes	NA	NA	Yes
Neil et al. [37]	NA	NA	NA	NA	Yes	NA	NA	Yes
Pelletier et al. [35]	Unclear	NA	NA	NA	Yes	NA	NA	Yes
Realpe et al. [32]	Unclear	NA	NA	NA	Yes	NA	NA	Yes
Sin et al. [28]	Unclear	NA	NA	NA	Yes	NA	NA	Yes
Terp et al. [30]	Yes	Yes	NA	NA	Yes	NA	NA	Yes

## Discussion

This review highlights the heterogeneity of co-produced research on psychosis in terms of the terminology and methods employed, as well as the quality of the research itself, the co-production element and reporting. Although the included studies reported service user involvement in multiple stages of the research process and emphasised its benefits, few described their methods of co-production in sufficient detail. Levels of involvement appeared to vary, from service user researchers conducting the research, to steering group members providing consultation and feedback, but with control of the research remaining largely in academic researchers’ hands. Further, several studies employed service users both as research participants and members of the research team; in these instances, it was challenging to distinguish between the service users’ roles, which may have resulted in their involvement being more tokenistic than co-productive.

In their critical analysis, Tierney et al. echoed this challenge: studies employing user involvement failed to provide a working definition of involvement, methodologies often lacked congruence, and although studies cited positive outcomes, it remained challenging to assess their quality and this raised questions about bias [43]. Importantly, in their choice of terminology, Edwards and Elwyn explained that authors do not always recognise the difference between involvement by shared information and involvement by shared decision-making power, incorrectly characterising their approach as “co-production” or similar [44].

Power-sharing difficulties are a key barrier to achieveing truly collaborative research: the equality of power necessary for co-production cannot be actualised while service users are perceived as having limited capacity [45]. Previous research has detailed that positive reports of service user involvement, such as effective communication, must be balanced with an acknowledgement of barriers, such as service users feeling overburdened by their involvement [46]. Co-production is also subject to social and economic constraints, as co-producers tend to be older, female and living in urban environments [47]. Studies included in our review emphasised turnover of staff, rather than the constraints faced by individuals with psychosis, as one of the biggest barriers, as lack of continuity has a significant impact on the patient/practitioner relationship as well as the translation of research findings into practice [31, 32, 38].

Previous literature has suggested that academic researchers experience challenges when conducting a co-produced project. These challenges include building and managing relationships, defining and adapting the project’s scope, and maintaining a professional identity [48]. Past studies have indicated that co-produced research typically favours academics with specific personality traits, such as generalists with good communication and conflict resolution skills, and a creative individuals who can be flexible while maintaining research integrity and rigour [49].

When considering co-production in a mental health context, researchers must be sensitive to the stigma, discrimination and general disempowerment experienced by individuals with mental health conditions in services and in society at large. In particular, researchers must consider the power dynamic that exists due to the legal relationship between mental health professionals and patients [50].

### Strengths and limitations

This scoping review utilised systematic methods for the identification, appraisal and synthesis of research in an area that has often been criticised for insufficient standardisation of terminology, processes and reporting. Further, it draws attention to an underrepresented group in co-produced research: people with lived experience of psychosis. The methods used in this study for a relatively small number of languages and databases, and for peer-reviewed journal articles exclusively, can be built upon for a more comprehensive and global systematic review of the grey and published literature from a wider range of sources (see Table 6 for recommendations).

**Table 6:** Summary of Recommendations

Recommendations for improving co-produced psychosis research • Improve guidelines for co-production, for example, by standardising language and assessment criteria for different approaches to involvement • Report characteristics of research participants as well as those involved in co-production • Formally evaluate the co-production component, reporting clear outcomes and lessons learned (i.e., barriers, facilitators, etc.) • Seek to target under-represented groups in co-production; for example, people with psychosis in LMICs • Pay attention to power differences, be mindful of mental health stigma (e.g., language etc.) and safeguarding concerns that may arise in the research • Support stakeholders buy-in by prioritising good communication (e.g., setting goals and guidelines at the start of the project) and encourage creative formats that allow for an iterative research study
Recommendations for reviews of co-produced psychosis research • Consider more robust methods of screening for co-production; for example, by incorporating both INVOLVE (2018) principles and features in assessing eligibility • Consider thresholds for involvement of people with psychosis, specifically; where diverse stakeholders are involved and/or characteristics are not reported, follow up with authors where possible and have a protocol for finalising screening decisions • Involve a multilingual advisory group with varied regional expertise in refining search terms and screen in multiple languages • Test search strategy to identify a parsimonious but inclusive set of search terms related to co-production • Include grey literature and incorporate additional sources of literature (e.g., hand searches, expert consultation, etc.) • Use double-screening (titles/abstracts/full-texts) and quality appraisal as well as data extraction, etc. to improve reliability

However, these methods are also subject to several limitations which must be taken into account: (1) first, as this is a scoping review, we prioritised sensitivity over specificity, and may have been over-inclusive in our search and selection process; (2) on the other hand, the age restriction employed in screening may have excluded potentially relevant studies, for example, on early intervention in psychosis; (3) the quality of reporting may limit the robustness of any conclusions that could be drawn; (4) lack of standardisation in the evaluation of co-production made it difficult to define outcomes; (5) more targeted methods may be required to correct for the over-representation of UK studies.

As described in our methods, we employed a broad definition of co-production in our eligibility assessments. Future reviews might consider whether to adopt both the INVOLVE principles and key features in screening decisions in order to reduce heterogeneity. After applying the key features during data extraction, we came to question whether several of the included studies reflected a co-production approach in practice, or just in principle.

Many studies were also insufficiently detailed, for example in describing the characteristics of the users involved in co-production as well as the research participants. Collecting and reporting this information is important not only for screening, but also as an indication of how representative a sample is and whether certain groups may be more or less likely to engage in co-production. Further, our eligibility criteria specified an age limit and a focus on psychosis. Clarification was sought from the authors; however, they were generally either unable to provide missing information or did not respond. Rather than automatically exclude these studies, screeners made judgment calls based on the best available information (for example, assuming that studies mentioning “adults” fulfilled the age criterion, even if mean age was not recorded). Subsequent reviews may be less generous in their screening decisions; however, as this is a scoping review, we felt it would be benenficial to be as inclusive as possible, in order to better understand the research landscape in this area. We would also suggest that future reviews consider removing the age restriction entirely, as this was not only difficult to apply in practice, but may have led to the exclusion of potentially relevant studies.

Inadequate reporting on methods made it particularly difficult to employ the Joanna Briggs quality appraisal tools, which focus on whether the research project is succinct, congruent and well-justified. The included studies usually involved several workshops, interviews, focus group discussions and questionnaires, often with small sample sizes and without any clear methodological justification. This presents a challenge not just for research synthesis, but for anyone seeking to reproduce a co-produced research study. Indeed, this scoping review highlights a common theme in the literature on co-production: one of the most critical challenges lies in the conceptual and methodological ambiguities for practical application [51]. There was no uniform approach to co-production across the studies included in this scoping review, which covered a wide range of different terminologies and methods, making it difficult to compare and contrast results as part of a robust synthesis.

Finally, it is important to note that this review is heavily skewed toward high-income anglophone countries—the UK specifically. A search strategy covering more involvement terms (e.g. “peer” and “stakeholder” involvement, etc.), more languages, more databases and a wider range of publication types—grey literature, in particular—could potentially uncover more co-produced research on psychosis from around the world. Involving a multilingual advisory group with expertise in different world regions would be especially helpful to improve on our methods. However, in the case of LMICs in particular, it is possible that there simply is not much relevant literature currently available. A 2016 review of user and caregiver involvement in strengthening mental health systems in LMICs identified just one example of user involvement in research (in Brazil), and this was limited to data interpretation [52]. An update published in 2019 identified another possible example in India, in which some data collectors with disabilities may have also had psychosocial disabilities, but this could not be confirmed from the text [53].

### Conclusion

This scoping review explored the peer-reviewed literature on co-production in psychosis research. The terminology and methods employed in this area of research vary greatly: even when the same term is used, the methods described may differ, and vice-versa. The quality of co-produced research is also challenging to assess, both because of poor reporting and the lack of methodological justification by researchers, and because co-production simply does not follow a traditional, linear research process. Co-production is often complex, involving multiple stakeholders working together through an iterative process. Yet, there are common barriers and facilitators to co-production with people with lived experience of psychosis. Unfortunately, although co-production is based on the ethic of shared power and equal collaboration, our scoping review suggests that this is not always the case in practice. Researchers should work to address these barriers and build on common facilitators from the earliest stages of study design, to improve the chances of successfully co-producing research with this under-represented population.

### Supplementary Information

**Additional file 1. **A list of the search terms used in the PsycInfo database.

**Additional file 2. **A extended version of the data extraction table (see Table 2) outlining study and participant information for the references included in the systematic review.

**Additional file 3. **An extended version of the data extraction table (see Table 4) listing the characteristics related to coproduction from the references included in the systematic review.

**Additional file 4. **A complete list of the INVOLVE principles and key features, which were used to support the data extraction and analysis process.

**Additional file 5. **A completed PRISMA checklist that details the steps taken during the systematic review.

## Data Availability

Not applicable.
