# Supplementary material for: Co-producing research on psychosis: a scoping review on barriers, facilitators and outcomes
Source: Int J Ment Health Syst. 2023 Aug 30;17:25. doi: 10.1186/s13033-023-00594-7 (PMC10466887; doi:10.1186/s13033-023-00594-7)
Supplement: Supplementary file 1 — Additional file 1. A list of the search terms used in the PsycInfo database. [file 13033_2023_594_MOESM1_ESM.docx]

## Appendix A: Search terms

*Example search terms from PsycINFO:*

(psychos#s or psychotic).mp. OR exp Psychosis/ OR exp Schizophrenia/ OR hallucinat*.mp. OR exp Hallucinations/ OR (percept* adj3 dis*).mp. OR exp Perceptual Disturbances/ OR delusion*.mp. OR exp Delusions/

AND

(co-produc* or coproduc*).mp. OR (co-design* or codesign*).mp. OR (co-creat* or cocreat*).mp. OR (user-led adj3 research).mp. OR (community-based adj3 research).mp. OR "participatory research".mp. OR exp client participation/ OR (patient adj3 public adj3 involvement).mp. OR (patient-led adj3 research).mp. OR "service user involvement".mp. OR "service user engagement".mp. OR "expert* by experience".mp.
