# Supplementary material for: Co-producing research on psychosis: a scoping review on barriers, facilitators and outcomes
Source: Int J Ment Health Syst. 2023 Aug 30;17:25. doi: 10.1186/s13033-023-00594-7 (PMC10466887; doi:10.1186/s13033-023-00594-7)
Supplement: Supplementary file 2 — Additional file 2. A extended version of the data extraction table (see Table 2) outlining study and participant information for the references included in the systematic review. [file 13033_2023_594_MOESM2_ESM.docx]

## Appendix B: Study and Participant Characteristics Extraction Table

| ***Reference*** | Country | Research Topic | Study Design | Sample Size | Recruitment | Participant Information | Diagnosis |
| --- | --- | --- | --- | --- | --- | --- | --- |
| ***Csipke, et al. (2016)*** | England | “service user and frontline staff perspectives on psychiatric ward design through participatory methods” | Descriptive  “the ‘‘SURE model’’ to elicit service user and staff perspectives on psychiatric ward design.”  Mixed methods model | Service users (N=63)  Nurse (N=10)  Staff (N=61) | Purposive sampling | Interview phase 10 service users and 10 nurses  Questionnaire phase 53 service users 61 staff were  36 service users participating in questionnaire agreed to take part in the photographic study | “Schizophrenia/psychosis Bipolar Disorder Depression/ Anxiety Substance Misuse Dual Diagnosis  Other Not Disclosed” |
| ***Higgins, et al. (2017)*** | Ireland | “EOLAS programme designed as a co-facilitated programme; with service users and family members bringing their personal knowledge and experience of living with mental health issues, and clinicians contributing their professional knowledge and expertise” | Descriptive  “Participative philosophy of collaboration” and “the principles of participatory action research (PAR)” | Participant (N = 58)  Service user (N= 30)  Family member (N=21)  Clinician (N= 7)  Discussion (N=6) multidisciplinary clinical teams.  Clinician (N= 13)  Peer facilitators (N= 10) | Recruited through “invitation by their key clinician” and “posters displayed in public areas in mental health facilities”  “ad hoc approach” | “nearly three-quarters were male (N=20, 74%) ranged in age from 23 to 80.”  “Approximately half schizophrenia”  “28% bipolar disorder”  “8% of participants had been diagnosed with schizoaffective disorder and the remaining  8% multiple diagnosis such as severe depression or psychotic depression”  “75% were female”, "ranged in age 31 to 70. Approximately three quarters of the sample were married”, “just under half had completed third-level education.” “majority were parents (40%), partners (30%) or siblings (25%). The majority of the sample were aware that their family member had received a specific diagnosis (most commonly schizophrenia).” | Schizophrenia and Bipolar Disorder |
| ***Kristensen, et al. (2018)*** | Denmark | Patient-reported outcomes – “the patient’s perspective to systematic monitoring of the quality of care and treatment and clinical quality improvement” | Descriptive  Co-creation | Steering Group (N=20)  Patient Peer Broad (N=10) | Not reported | Not reported | Depression and Schizophrenia |
| ***Larkin, et al. (2015)*** | England | Describes translation of “three qualitative research studies about hospitalization in early psychosis (exploring the perspectives of service-users, parents, and staff) and translated them into service improvements developed in collaboration with a range of stakeholders, including service-users, carers, community and inpatient staff, and management.”  Outlines process with “Experience-based co-design (EBCD)” | Qualitative  EBCD | N= over 150 stakeholders in N= 20 feedback groups,  Co-Design Event  (N=3) EIS-users and (N=2) family members from the “patient experience” and clinical research teams.” | Purposive sampling | Not reported, simply identified as “young adults” | Psychosis |
| ***Morant, et al. (2018)*** | England | Explored “service users’ experiences of taking antipsychotic medication for psychotic disorders and their perceptions of decision-making about this.” | Qualitative | (N= 20) | Purposive sampling | 13 males, 7 females  “Schizophrenia or schizoaffective disorder”  “Wide range of self-reported duration of antipsychotic”, “half the sample reporting more than 10 years of antipsychotic use.”  “Two participants were subject to Community Treatment Orders”, a legal measure | Schizophrenia and Schizoaffective Disorder |
| ***Neil, et al. (2013)*** | England | “Service Users and researchers worked together to develop a measure of recovery from psychosis the Questionnaire about the Process of Recovery from psychosis” | Descriptive  Report describing coproduction methods from another study Neil et al., 2009 | (N= 10-15) Service Users, with personal mental health provided “expert consultation” | Convenience sampling | Not reported | Psychosis |
| ***Pelletier, et al. (2015)*** | Canada | “Explore the feasibility and acceptability of patient partnership for developing an interactive guide to improve access to primary care providers for chronic diseases management and health promotion among patients with severe mental illnesses” | Descriptive  Quantitative  Participatory action research design | (N=146) participants | Recruited through IUSMM Archives Department | “95 males (65.1 %) and 51 females (34.9 %)”  “Mean age is 52.7 years old with a standard deviation of 14.8 years.” | Schizophrenia |
| ***Pitt, et al. (2007)*** | England | “User-led research looking at recovery from psychosis from a service user perspective” | Qualitative | (N=7) | Recruited through mental health groups | 5 males, 2 females  18 to 65 years  6 White and 1 mixed race. | Psychosis |
| ***Realpe, et al. (2019)*** | England | “Co-designing a virtual world with young people to deliver social cognition therapy in early psychosis” | Descriptive  Iterative co- design process | Stage 1&2: 2 service users Stage 3: four service users  Stage 4: four volunteers from target population but without mental health history  Stage 5: 20 participants recovering from first episode psychosis | Recruited through mental health partnerships and universities | “Mainly resided in UK cities and were engaged on higher education or work placement schemes. Service user volunteers were actively involved in mental health advocacy and research.” | Psychosis |
| ***Roelandt, et al. (2020)*** | Algeria, Canada, France, Greece, Hungary, India, Italy, Lebanon, Lithuania, Madagascar, Mauritania, Mexico, Morocco, Spain, and United Kingdom | Protocol for “international participatory study implemented in 15 different countries focused on “depressive episode” and “schizophrenia”, examined whether service users and carers reported understanding the diagnosis, whether they associated it with a negative feeling, whether they would prefer rephrasing it, and in which terms, and whether they used the label when talking with family and friends” | Cross-sectional  Mixed methods | “279 users and 232 carers for "depressive episode" and of 263 users and 255 carers for "schizophrenia"” | “Recruited as they visited the mental health services” | “Users and carers were similar in terms of level of education, but differed on other criteria.  Compared to users, carers were more often female, older, more often employed and less often single, alone, and childless.” | Schizophrenia and Depression |
| ***Schneider, et al. (2004)*** | Canada | “Communication Between People With Schizophrenia and Their Medical Professionals: A Participatory Research Project” | Qualitative  Participatory research | (N=11) | Not reported | Not reported | Schizophrenia |
| ***Sin, et al. (2019)*** | England | “Integrate participatory research methodologies by the public, patients, and carers into the eHealth (electronic health) intervention design and build process to improve the product’s usability and acceptability.” | Descriptive  Participatory Research Methodologies | Total carers (N= 24)  First alpha-build (N= 10)  Second alpha version (N=14) | Recruited EAG from various “clinical and voluntary service provider organizations” “Members were paid a goodwill payment for meeting attendance and contributions and were also reimbursed for their travel expenses according to the INVOLVE payment scheme”  “Carers independent from the study were consulted on the evolving drafts of the intervention prototype through focus group meetings.” | Carers  10 men and 14 women  ages between 22 to 83 years  Retired (n=9)  At home full-time carer (n=3)  Working full time (n=5)  Working part time (n=6)  Actively seeking employment (n=1).  A parent (79%), spouse (8%), a close friend (8%), an adult child (4%).  Carers lived with their cared-for person during the study (38%).  Cared-for persons  11 male, and 13 females, between 17 to 61 years  Half had a diagnosis of psychosis, 9 were diagnosed with a schizophreniform disorder and 3 had bipolar disorder (type 1) | Psychosis or Schizophreniform |
| ***Susanti, et al. (2020)*** | Indonesia | “Part of a larger development award exploring the potential of involving patients, carers and communities to strengthen mental health systems in Indonesia” “aimed to develop a culturally appropriate PPI framework” for use in Indonesia for “strengthening local mental health systems” | Qualitative | PPI advisory group (N= 12)  Participant (N= 43) with  (N=22) service users and (N=21) carers | Convenience sampling | Service users  17 males, 5 female, average age 38.8  Carers  5 males, 17 females, average age 51.7 | Psychosis |
| ***Terp, et al. (2016)*** | Denmark | Reports the co-design process, that “enables young adults with schizophrenia to become active participants in the design of a more participatory mental health practice” | Qualitative  Participatory design | (N= 14) | Purposive sampling strategy “ | 7 young adults, age 19–31 years diagnosed with schizophrenia  7 healthcare professionals | Schizophrenia |
| ***Tischler, et al. (2010)*** | England | “collaborative study with service users focusing on the concept of patient-centredness, which attempted to be as patient-centred as possible in terms of the process and outcome of the research  “explore the experience of both professionals and patients taking part in a research project that strove to be collaborative and patient-centred” | Mixed methods community study | Mental health service users(N=14)  Senior psychiatrists (N= 3) Psychiatrists/mental health service user (N=1) | Purposive sampling | Not reported | Schizophrenia |
