# Supplementary material for: Co-producing research on psychosis: a scoping review on barriers, facilitators and outcomes
Source: Int J Ment Health Syst. 2023 Aug 30;17:25. doi: 10.1186/s13033-023-00594-7 (PMC10466887; doi:10.1186/s13033-023-00594-7)
Supplement: Supplementary file 3 — Additional file 3. An extended version of the data extraction table (see Table 4) listing the characteristics related to coproduction from the references included in the systematic review. [file 13033_2023_594_MOESM3_ESM.docx]

## Appendix C: Co-production Extraction Table

| ***Reference*** | Terminology | Methods | Barriers/ Facilitators | Outcomes |
| --- | --- | --- | --- | --- |
| ***Csipke, et al. (2016)*** | ‘‘SURE model’’ is a participatory methodology | “SURE model- participatory methodology premised on collaboration with service users across all stages of a study”  “two service user researchers surveyed the literature, performed the data collection and undertook a considerable part of the data analysis.”  “one service user researcher had experience of the service under investigation”  Provided feedback to reach consensus during co-design, film shown and participants co-designed service improvements  “Interviews were audiotaped, transcribed and thematically analysed by two service user researchers independently”  “The free text responses on the two questionnaires were collated and thematically analysed by a service user researcher.” The service user researchers also administered the photography component of the study | “camera might facilitate recruitment by making participants feel more involved in the process of research, allow for the negotiation of language barriers, and provide us with more in-depth understanding of service user experience of the wards” | ““user generated tools [..] may enable an in-depth assessment of the experience of in-patient care for service users and frontline staff alike” |
| ***Higgins, et al. (2017)*** | Participatory action research (PAR)  “focusses on doing research ‘with’ people as opposed to ‘on’ people” | “Fostering ethos of self-determination, co-creating and co-translating new knowledge into action for change”  “Action research group involved service users, family members, clinicians from a range of disciplines (medicine, nursing, social work and psychology)”  “co-ordinated by a steering group comprised of representatives from all the stakeholder groups involved.”  “Core values agreed at the outset […] Support, Understanding, Partnership, Participation, Openness, Respect and Trust”  Subcommittees responsible for “various aspects of project development, design, recruitment and roll out.”  “Focus groups held with service users and family members”  “Separate handbooks with information pertaining to each session were developed for service users and family members to accommodate the diverse needs and preferences of service users and family members”  “Each session comprised input from the facilitators, learning activities” “Other people from the local mental health service […] were invited to provide input” | “generate ‘buy in’ clinicians were also invited to attend group discussions, and consultation meetings were held with multidisciplinary teams.”  “absence of any guidance within the literature and in recognition of the challenges of developing co-facilitation skills” | “ Flexibility of the interview would shed light on unexplored or previously unexamined perspectives, such as the challenges of peer-clinician  co-facilitation, the value of peer involvement and of programme handbooks” |
| ***Kristensen, et al. (2018)*** | Iterative co-creation process | “Patient involvement lies at the heart of this study, output based on iterative co-creation process between patients and healthcare professionals”  “PPB operated through workshops, on their own, dialog based in pairs and in the group to set priorities and reach consensus recommendations”  “The SG and PPB worked separately at the request of the PPB in an iterative co-creation process”  PPB commented on  Data collection  Patients' information requirement when answering PROMs  Graphical display of the PROMs for data collection  Graphical display of PRO results for self-management” “19 items co-created and/ or selected by the PPB and the SG | “PRO-Psychiatry’ builds upon a mix of methods leading to consensus output between patients and clinicians”  “ accounted for political, cultural, professional, and patient issues, which were brought to light and handled dur- ing the consensus process”  Organization “allowed patients to be directly and indirectly involved at all project stages and an opportunity to voice their opinions on a larger variety of issues”  “Emphasis on direct patient involvement is likely to build trust that the PRO-data will not be misused, which is a pre-condition for successful application” | Task of the PPB was to voice the patients’ opinions”  “Emphasis on direct patient involvement is likely to build trust that the PRO-data will not be misused”  Patient Peer Board successfully “emphasized concrete, unambiguous easily understandable information, and procedures for data collection and display of results” |
| ***Larkin, et al. (2015)*** | “EBCD is a collaborative approach that aims to improve health care services by enabling service-users, carers, and staff (ground-level and management) to collaborate together to co-design better services” | “improve health care services by enabling service-users, carers, and staff (ground-level and management) to collaborate together to co-design better services”  “sequence followed by EBCD projects has evolved with the literature, but tends to follow a basic process of (a) gathering experiences from staff, then service-users and carers, via observation and interviews (which are often filmed); (b) identifying “touchpoints” (critical moments experienced in relation to the service) and (c) feeding these back to the project participants; (d) prioritizing the touchpoints by the project participants; (e) bringing everyone together in a co-design event, where they work in small groups to co-design improvements to the service according to the priorities identified; and (f) holding a celebration event to allow all involved to review what has been achieved” | “Challenges that are generic to EBCD”  “organization was being restructured, and a number of our collaborators and nominated “champions” either left or changed role, and could no longer work with us”  Research and co-design phases  “once we had handed over to the steering com- mittee, the lack of continuity in high-level support meant the responsibility for making improvements fell on a small group of individuals who were increasingly under pressure and had little “power” when it came to imple- menting change. An EBCD project relies on a great deal of good will, commitment, and trust between all parties, and having people both internal and external to the ser- vice in our team certainly helped project management.”  “Mechanisms to encourage ownership of the project, as well as strong high-level support, are essential to guarantee the implementation and sustainability of improvements.”  “Strategic, budgetary, or staffing commit- ments.”  Additional Challenges  “EBCD being seen as an additional burden in an already pressurized”  “when co-designed improvements were not implemented, for whatever reasons, participants were left feeling disappointed and dissatisfied”  Concerns Specific to the Mental Health Context  Safeguarding “Safety procedures were included, such as contacting participants’ care teams if they were distressed by their participation in interviews. “  “choosing what material to share at the co-design event, even in the form of anonymous quotations from the original interviews, was also a matter for careful con- sideration. While not wanting to “censor” personal account”  Importance of formal feedback and ongoing commitment from the steering group  “External research staff may not face the same issues gaining the trust of service-users and families, but their lack of local knowledge is likely to be detrimental in setting up, managing, and implementing the project”  “we would advise either a close relationship with an inter- nal partner (as we had) or for the researcher to be embed- ded, at least temporarily, within the service-environment.” | All feedback groups reached consensus fairly easily and recognized the typical difficulties faced by young service-users being hospitalized”  “Attendance at the steering group was consistent among a small group of committed staff, but there was frustration at the lack of time and organizational support available to follow through the plans”  Authors conlcude “ it necessary to develop and adapt the method to suit the vulnerable populations and complex services we were working within, but we feel able to con clude that it is feasible to use EBCD in a mental health context, provided careful attention is given to ethical and safety issues” |
| ***Morant, et al. (2018)*** | “To explore service users experiences of taking antipsychotic medication for psychotic disorders and their perceptions of decision-making about this” | “semi-structured interview schedule was developed in collaboration with an established mental health service users group South Essex Service User Research Group( SE-SURG) over the course of three meetings”  “ Interviewers disclosed their status as mental health service users at the beginning of the interview”  Awareness “reconceptions as academics, research- ers and clinicians and integrated service users perspectives into early stages of the analytic process: Service user interviews were invited to review discuss and modify an initial set of emerging themes in the half-day meeting, with prior preparation” | “Enhance rapport and openness, interviews were conducted by five service user researchers from SE-SURG” | In depth exploration of service users views of their involvement in decision-making about antipsychotic medication. As well as revealing the impacts of taking antipsychotics, it highlights how experiences of medication decision-making are characterised for many by passivity, perceived limitations of choice, low levels of involvement, and a sense of powerlessness” |
| ***Neil, et al. (2013)*** | Collaboration, Service user researchers | “Collaboration between a trainee Clinical Psychologist (STN), three Clinical Psychologists and two Service User researchers with personal experience of psychosis.”  “Monthly research supervision”  “Consulted a Steering Committee on four occasions, from planning through to dissemination of the study.”  Developing the research question  “Individuals with research methodology experience […] supported others to understand what measures are used for and how they are developed.”  Individuals with “personal experience of completing measures supported those without, to consider potential strengths and difficulties related to being asked to complete measures whilst one is experiencing psychosis.”  Researchers  “Acknowledged the measure potentially did not incorporate all of the Service Users’ views on the final items, which they considered important, as they were keen not to engage in tokenistic consultation.”  Naming the measure  “Discussion took place on the name and whether or not the word psychosis should be in the title as some Service Users might find this term stigmatising.”  “As the study progressed, and on its completion, the research and the findings were disseminated through presentations at special interest groups and amongst peers, both by Service Users and non-Services User researchers. Service Users employed dissemination strategies (e.g. discussion with peers at Service User forums), which might have been inaccessible to some Service Users and staff. The involvement of Service User consultants in dissemination potentially resulted in the findings being accessible to a wider audience that moved beyond publication in an academic journal.”  “we wanted to disseminate, the different journals we could potentially publish in, who our target audience was and who the authors would be. It was mutually agreed that one paper should focus on the development of the QPR and a second paper should be written up with a focus on collaborative research. Service Users unanimously expressed that STN should lead on the papers as the original study was part of her doctoral thesis, how- ever. It was mutually agreed that Service Users and Clinical Psychologists would be co-authors and non-Service Users were very keen for as many Service Users as possible to be involved in the write up of each paper. Service User researchers and Steering Committee members also advised on the use of non-stigmatising language for the papers.” | “Service Users was positive towards developing a measure of recovery. Several individuals were, however, also apprehensive (based on their own experiences) around the potential for its “misuse” and expressed concerns about the QPR possibly being “harmful” to Service Users.”  “First, researchers acknowledged, that during the research phase of the study, the research questionnaire would not be imposed on anyone, that is, participants would need to provide informed consent, and would also be informed that they could con- tact the researchers if they became distressed during the study. Second, the wording used in the instructions was designed to reduce the potential to cause distress to Service Users. Third, it was suggested that during the study, participants would be asked whether the QPR was distressing to complete, using a visual analogue scale. It was agreed that if the results indicated that participants were distressed by the QPR then we could decide at a later date that it would not be published for use in the public domain. Finally, it was agreed that should we publish the questionnaire in the future, further safeguards could be put into place to prevent its misuse, that is, copywriting the measure and writing specific instructions for professionals administering the QPR. The above solutions were acceptable to everyone and were implemented.”  Challenges during data collection “Comments included that the questionnaire pack was “daunting” and that one of the questionnaires was “difficult” and that peo- ple could find this “even more difficult” if they were not “feeling well.” The Ser- vice Users involved, advised that the questionnaire pack could be condensed and to emphasise that people did not have to complete all the questionnaires if they did not feel able to, that the following sentence be added to the front sheet; “If you find you are not able to complete them all, please fill out as many as you can.” These suggestions were implemented and positively impacted on recruitment.”  “Although every effort was made to avoided tokenistic involvement, and discussion around data analysis did take place with all contributors, it is acknowledged that Service Users could not be completely involved in the statistical analysis as the study was part of my doc- toral thesis.” | “User with experience of psychosis, and some with experience of completing questionnaires. By working together and sharing our areas of expertise, for example, research knowledge and expertise by experience, we produced a recovery measure, which is meaningful and valid.” |
| ***Pitt, et al. (2007)*** | “ ‘user-led research’ refers to research where service users control all stages of the research process, including design, data collection and analysis, writing-up and dissemination” | “ two user researchers (L.P. and M.K.). They met regularly with a steering committee of further service users and together they made decisions about the topic and design of the study, and conducted all stages of the research into recovery from psychosis. Research supervision was provided by clinical psychologists”  “steering committee of service users was set up to decide the topic, provide guidance on the design of the study and have input into the analysis.”  “ interpretative phenomenolo- gical analysis (IPA), which is a form of analysis that is particularly suited to the exploration of subjective experience. It is concerned with the participants’ percep- tion of their experience and the sense they make of it themselves.” | “importance of promoting and encouraging active participation by service users and the need for strengthening key relationships with professionals who are able to engage in active dialogue with the service user.”  “ empowerment as a key theme and supported the views of Tew (2002), which are characterised by a collaborative working relationship built on trust, respect and equality.”  “attendance can induce considerable anxiety in certain individuals and every attempt should be made to minimise their distress.”  “choice of venue, providing a comfortable environment that is large enough to accommodate all those present without being too cramped. The service user should be advised beforehand that they can bring along an independent representative of their choice. This could be a family member, a close friend or a colleague. In addition, practitioners should also make every effort to ensure sufficient time is given to all parties present. Any points made and raised by those present should also be fed back to the contributor to ensure clarity and respect of an individual’s opinion.”  “ Unfortunately, within mental health services there is not only a rapid turnover of staff but also in some instances expectations of practitioners that service users will be seen by many different doctors”  “importance of meaningful key relationships as highlighted in this study, this inconsistency may not progress such an important aspect of care” | “Importance of promoting and encouraging active participation by service users and the need for strengthening key relationships with professionals who are able to engage in active dialogue with the service user” |
| ***Pelletier, et al. (2015)*** | “Participatory action research (PAR) is an approach that involves study participants in all aspects of the process, from conceptualization to data collection, through interpretation and dissemination of findings.” | “The research team followed as strictly as possible the development process that was implemented in Australia by the Queensland Government”  “patients are also actively involved in such a process as research partners throughout (patient research partners). For instance, this project was coordinated by patients who are members of the International Program for Participatory-Action Research (IPPAR)”  “The goals of small group learning include the following: 1) to reinforce knowledge through problem solving; 2) plumb the depths of a problem; 3) test assumptions; 4) generate hypotheses and practice critical reasoning; 5) collaborate with peers, and; 6) to receive feedback” | “ Other potential barriers to effective implementa- tion are the perceptions of incompetence, of danger- ousness, and of permanent impairment that health care providers, like the public in general, can have towards patients with SMI”  “The active and visible participation of patients in the small group sessions among health care providers should help to generate a more positive image and to strengthen GP’s confidence in engaging themselves in health promotion dialogue with SMI patients.”  "Small group learning is an educational approach that allows participants to develop problem solving, inter- personal, presentational and communication skills that are difficult to develop in isolation, and require feed- back and interaction with other individuals.” | “The patient-centeredness of this participatory research was characterised by an enhanced level of patient commitment to this R&D process of the IGMA and its use”  “Difficult communication and social distance have been identified in the literature as bar- riers to equitable access to primary care providers and this access can be improved through the use of a tool like the IGMA and the interactivity and collective social support that its use generates among small groups of patients” |
| ***Roelandt, et al. (2020)*** | Persons with lived experience | “Participation of person with lived experience”  “Interviewers had to be health professionals working in the field of mental health, including peer workers and experts by experience.”  “Users and carers were involved in all the stages […] they participated in the development of the protocol, in the review of the design and the materials, in pretesting, and in the interpretation of the results.” | “shortcomings, related to the design itself, but also due to the construction and the international character of the ICD”  “all users had their diagnosis disclosed. It implies that professionals commu nicated at least that one impactful piece of information. Knowing that not all people are given their diagnosis, results and their implications may not be applicable to persons not informed of their diagnosis or outside of the health care system” | “Revealed a gap between official medical language and users’ and carers’ language.” Experiential knowledge “could be a source of relevant information to reduce the gap between chosen terms and underlying concepts” |
| ***Realpe, et al. (2019)*** | Co- design | “The co-design process involved five stages centred in key design questions”  “We discussed each stage with young service users and used their suggestions to advance to the next stage. The stepped approach was based on the methodological guideline for the implementation of participatory design of online treatment for young people” | “Young people highlighted important issues in the design and were empowered to influence the development of the intervention. How- ever, setting up these groups required considerable time and effort and came with caveats (eg, continuity). Our struggle reflects a lack of opportunities and support systems to facilitate collaboration between young people and researchers in developing research together, and this needs further attention in research environments. Meaningful input from user and carer consultants needs to be part of any study.”  “Service users warned about perceived barriers to the success of the study, focusing on accessibility to the technology. One focus group participant wondered about how the research team would address potential “limited accessibility due to requirement of computer and internet.”  “Finally, young service users suggested study participants would require comprehensive information about data security in relation to access to the environment and data storage.”  “Be clear about security of data storage – who has access to the data, etc. this may be a particular issue for people with a history of paranoia/psychosis.” | Participants in this study challenged researchers' understanding of what young people need during recovery”  Findings contribute to current efforts to develop environments that reflect lived experience.  “The process of co- design led to the development of a specific approach and protocol to be tested in a proof-of-concept trial with people experiencing a first episode of psychosis”  " Service users supported novel methods of engagement of people who may be suffering in isolation and found virtual worlds to delivery therapy acceptable”  “The co-design process permitted a feedback loop that continues to inform design and solve problems as they emerge in the pilot study” |
| ***Schneider, et al. (2004)*** | “Participatory research involves members of the research group in meaningful participation in all stages of the research process.” | “Group members chose the topic” and the “method of data collection”  Research assistant “helped analyze the interview transcripts”  “Group members produced a set of recommendations for how they would like to be treated by their medical professionals. Results of the research were disseminated to the public in the form of a readers’ theater presentation developed and performed by group members […]  main conclusion of this participatory research project is that good communication with medical professionals is essential in the lives of people with schizophrenia”  "discussion of the benefits of this project in generating knowledge about the experiences of people with schizophrenia, in offering a transformative experience for the people involved” | “Good communication”  “The script was written by the university researcher based on group suggestions about content and includes quotations from the inter- views selected by group members. The group’s very powerful and moving presentation has, at this writing, been performed seven times and been seen by several hundred health care professionals. To achieve wider dissemination of the project through academic publication, the university researcher asked permission from the members of the group to write an academic article and to include them as coauthors. Written by the “lead researcher, drawing on academic literature in the field, material generated in the research, and discussions among the participants. However, as the principles of participatory research remind us, true participation belongs to those who take part, not those who write about them.” | “This research project empowered one small group of very marginalized people with schizophrenia to speak directly to psychiatrists and other mental health professionals about their treatment experiences and through this is contributing to change in how others with mental illnesses are treated by their health care professionals” |
| ***Sin, et al. (2019)*** | Coproduction, co-design | “co-design and build work were directed by an expert advisory group (EAG) comprising individuals and carers with lived experience of psychosis and professionals working in health, social, or voluntary sectors with the target population.”  "Used the coproduction in mental health improvement work method to organize the workshops” (Iterative Consultations  and Coproduction Workshops)  “emphasizes and values the hidden capacity and capability of end users, families, and significant others”  “each workshop, all members assumed equal decision-making role, whereas we contributed with our respective strengths and expertise”  “These participatory design workshops (and follow-up work) were mapped to fit the 5-step development process of eHealth products”  “After each participatory design workshop, the knowledge and ideas generated were translated to produce draft hand-sketched plans and wireframes, mockups of Web pages, and source materials for the intervention”  “Mockups and output produced between workshops and end users’ feedback obtained from consultations”  “The EAG decided the master plan for the content and key functions of COPe-support” | “EAG believed that the testimonials from other carers who helped develop the intervention would add credibility to the intervention; hence, these testimonials and photos of the EAG working together were added to the website”  “EAG reviewed the essential ingredients as identified by our earlier development work [,,,] to produce the draft architecture and mockup of shell.”  “nondigital design tools such as magic sheets, post-it notes, index-cards with content items written on them, papers, and pens to encourage a creative design atmosphere involving all members, regardless of their competency level of information and communication technology. EAG members did hand-drawn sketches of Web pages and organized the content and ingredients in a structure that they saw fit” | Authors commented that a significant outcome was the “documentation of a rigorous and innovative build process” |
| ***Susanti, et al. (2020)*** | Patient and public involvement (PPI) | “Develop the proposal with local collaborators “  “Two PPI consultation events were conducted during this trip with people with psychosis and their carers to inform the study design”  “choice of data collection method was informed by the study PPI advisory group.”  “PPI advisory group consisted of 12 people who either had lived experience of psychosis or cared for someone with a diagnosis of psychosis recruited through a partner non-governmental organization (NGO).”  “Advisory group was established at the initiation of the wider project and consulted on all project components.”  FGD’s “were enhanced through the inclusion of a carer-researcher who co-facilitated focus groups and contributed to the analysis of transcripts.” | Previous experiences “had been met with punitive responses from health professionals” e.g. “complaints were interpreted as a relapse in their condition”  “Involvement activity did not always bring about desired changes”  “Sharing burden, sharing skills and experiences, improving confidence and combating stigma”  “Important for their recovery as it gave them a sense of purpose and helped them to reintegrate into the community.” Carers “felt strongly that PPI should not overburden family members.”  “inclusion of senior academics and health professionals as co-applicants and lead researchers who could drive progress in Indonesia, the delivery of a research methods training course at study outset which was delivered to both Indonesian researchers and PPI contributors and regular supervision by Skype and during study visits”  “Unable or unwilling to engage in involvement activities for a range of reasons.” Thus offer a “range of involvement activities so individual engagement could fit with personal circumstances. Community organizations were considered best placed to offer such activities”  “a lack of resources to access services and already being burdened by longterm caregiving duties.”  Community members hesitation due to stigma  “Facilitate engagement from patients and carers, involvement activities should be offered to people which were distanced from their locality to avoid identifying”  “Professional capacity to implement PPI”  “perceived lack of relevance to their roles”  “Paternalism”  “improve their skills in delivering information, communicating with service users and carers”  “Lack of organizational readiness to implement PPI”  “poor co-ordination” “inadequate distribution” of resources, “low political salience of mental health services at a national level”  “lack of knowledge”, “lack of understanding about the benefits of involvement”  “need for national resources and guidance related to PPI in line with that developed by INVOLVE in the UK” | “Participants acknowledged a number of benefits of PPI including sharing burden, sharing skills and experiences, improving confidence, and combating stigma” |
| ***Terp, et al. (2016)*** | Co-design | “Design artefacts were used to support active participation” e.g., “storyboard, card sorting, mock-ups, paper prototypes” a metaphor “becoming the captain of your own life” | “Appropriate ways of involving and engaging people in activities […] used co-design […] workshops to establish participation.”  “Approach to design is emphasized as a means to seed positive social connections between participants, to immerse participants and to build a shared understanding about an issue that can be used as a basis to collaboratively generate, explore and evaluate new ideas or alternatives”  “success of a design process as a notion of a CoP might inspire individuals to participate and foster engagement”  “A sense of community”  Communication “important recruitment strategy” and “strategy to promote engagement in the early phase” | Initial phase of design development created “ownership and pride”.  Advantage of experienced participant-facilitated “construction and maintenance of a secure and informal environment that supports mutual engagement” |
| ***Tischler, et al. (2010)*** | Participatory research methods | Two of the psychiatrists “spent several months developing informal links and opening a dialogue about collaborative research with mental health service user groups”  “Facilitate an assessment of convergent validity, the service user group developed its own definition of patient-centredness as applied to research practice.”  An “iterative process,” where “service users devised a novel approach whereby each of the 13 participants selected a factor that they considered best reflected a patient-centred approach.” | “service users’ relative lack of research experience meant that it became more difficult to work in a truly collaborative manner. With support from the study team, the service users contributed to the design, changing several elements, for example removing jargon from the research abstracts constituting the stage 1 data source.”  “They raised the need for service users to have adequate support when attending research meetings with other health professionals, so that they are able to express their views without feeling intimidated or patronized, and for there to be a recognition that service users might require more support when undertaking research than ‘professional’ researchers.”  “Strong view that research publications should be written in a way that is understandable and accessible, and that research should address issues that service users view as directly relevant to them. The findings of stage 2 of the study indicate that both service users and professionals found the experience of participating in a collaborative study to be positive”  “It highlights the potential benefits of collaborative projects for both health professionals and service users despite the need for substantial resources and commitment to undertake such research.”  “power differential that exists between researchers and service users”  Motivated participants might have been “achieved through respecting and involving participants, who were highly committed as a result.”  “Although one service user was involved in the preparation of this paper, training and support could have been provided for this individual to foster equality of input and active participation in research.”  “both doctors and service users, experienced participating in the present study as valuable, enjoyable and meaningful”  “Genuine and meaningful, there may have to be considerable changes to conventional research development and implementation so that service users are involved throughout the research process as collaborators” | “Emphasizes the importance of the process of research in achieving genuine service user involvement. It highlights the potential benefits of collaborative projects for both health professionals and service users despite the need for substantial resources and commitment to undertake such research” |
