# Supplementary material for: Co-producing research on psychosis: a scoping review on barriers, facilitators and outcomes
Source: Int J Ment Health Syst. 2023 Aug 30;17:25. doi: 10.1186/s13033-023-00594-7 (PMC10466887; doi:10.1186/s13033-023-00594-7)
Supplement: Supplementary file 4 — Additional file 4. A complete list of the INVOLVE principles and key features, which were used to support the data extraction and analysis process. [file 13033_2023_594_MOESM4_ESM.docx]

## Appendix D: INVOLVE Principles and Features

INVOLVE (2018) Key Principles [6]:

**Sharing of power:** “the research is jointly owned and people work together to achieve a joint understanding.”

**Including all perspectives and skills:** “make sure the research team includes all those who can make a contribution.”

**Respecting and valuing the knowledge of all those working together on the research:** “everyone is of equal importance”
**Reciprocity:** “everybody benefits from working together”

**Building and maintaining relationships:** “an emphasis on relationships is key to sharing power. There needs to be joint understanding and consensus and clarity over roles and responsibilities. It is also important to value people and unlock their potential.”

INVOLVE (2018) Key Features [6]:

**Establishing ground rules:** “Establishing ground rules at the beginning of the project can help create an environment where all voices can be heard and treated with respect. These ground rules, developed by the group working on the research, would set out expectations, in terms of the roles, responsibilities and behaviours of all.”

**Ongoing Dialogue:** “There should be dialogue between all those working together on the research project. This dialogue should begin prior to the start of the project, to help identify different types of knowledge, roles, responsibilities, expectations and establish relationships.”

**Joint ownership of key decisions:** “It is the ‘joint ownership of key decisions’ which helps differentiate co-producing from collaborating. It is not that everyone needs to be involved in every decision or every aspect of a piece of research but rather that the group, working together, decide and agree who should be involved and when, in terms of the management, governance and undertaking of the research.”

**Commitment to relationship building:** “Embracing and embedding the principles really requires a coming together of the organisations which host research projects and the communities within which they exist. Addressing power differences and developing relationships requires the development of open, honest, trusting and reciprocal relationships. Trying to create a level playing field and emphasising the development of relationships have implications for both the culture and processes and procedures of organisations – many of which will take time to implement. Co-production won’t ‘just happen’. Organisations and researchers need to shift from being not just ‘doers’ of research but to being proactive in encouraging and facilitating public involvement and developing relationships beyond the research community.”

**Opportunities** **for personal growth and development:** “There is an emphasis on supporting individuals and unlocking the potential of individuals to contribute to the project. In this way people are treated as assets with the skills, knowledge and experience to help develop solutions to issues. Project leads need to facilitate the involvement of the public effectively and manage the flexibility and uncertainty that are often involved in co-produced research projects. Members of the research team need to be willing to relinquish power and accept reciprocity of experience and expertise. This may require a cultural change in the research team and/or the organisation hosting the team.”

**Flexibility:** “A research project usually has a pre-determined project plan. However, a co-produced research project should provide opportunities for an iterative, fluid, open ended, experimental and interactive process; there should be opportunity for solutions and innovations to emerge from the relationships developed.”

**Valuing and evaluating the impact of co-producing research:** “It is as important to value the impacts of working co-productively that come from the research process as it is of the research findings or outputs. For the research process, some of these impacts will emerge rather than be planned: new relationships, expanded social networks and increased confidence of members of the public may be some examples. In terms of the research findings or outputs, working co-productively will produce knowledge and an end result that will often be different from that produced by a conventional academic process.”

**Continuous reflections:** “Reflection is a process whereby research team members have the opportunity to look at and reflect on how they are working together, how they might be using their particular expertise and perspective in the project and how this might impact on the research process and findings/outcomes.”
